# Comparison of Alcohol-Induced Hepatoprotective Effects of the High Fischer Ratio Oligopeptides with/Without Half Substitution by *Pueraria lobata*

**DOI:** 10.3390/foods14223859

**Published:** 2025-11-11

**Authors:** Yongke Deng, Qin Zhao, Na Chen, Zhiqin Zhang, Jingxuan Wang, Hongbing Fan, Haimei Liu, Lili Zhang

**Affiliations:** 1 School of Food Engineering, Ludong University, Yantai 264025, China; 2024121064@m.ldu.edu.cn (Y.D.); lduzhaoq@126.com (Q.Z.); 13515358735@163.com (N.C.); 2023120984@m.ldu.edu.cn (Z.Z.); 2024121073@m.ldu.edu.cn (J.W.); 2 Department of Animal and Food Sciences, University of Kentucky, Lexington, KY 40546, USA; hongbing.fan@uky.edu

**Keywords:** high Fischer ratio oligopeptides, *Pueraria lobata*, alcoholic liver injury, antioxidant activity, hepatoprotection

## Abstract

Long-term excessive intake of alcohol can cause serious damage to the liver, and the study of natural active ingredients with hepatoprotective effects is of great significance for the prevention and treatment of alcoholic liver injury. This study explored the ameliorative effects of high F-value oligopeptides (HFOPs) from *Chlamys farreri*, *Pueraria lobata* extract (PL), and their complex (HFOPs + PL) on alcoholic liver injury. Results showed HFOPs + PL significantly reduced alanine aminotransferase (ALT) and aspartate aminotransferase (AST) leakage, increased superoxide dismutase (SOD) and glutathione (GSH) activities, and decreased the production of malondialdehyde (MDA) and inflammatory factors in alcohol-induced HepG2 cells. In mice, it prolonged intoxication time, shortened detoxification time, enhanced hepatic ADH and aldehyde dehydrogenase (ALDH) activities, reduced serum AST and ALT levels, and improved antioxidant capacity. Its effects were better than PL alone and comparable to HFOPs alone. HFOPs and PL alleviate alcoholic liver injury by enhancing ethanol metabolism, reducing oxidative stress, and suppressing inflammation, providing theoretical support for their combined use in alcohol detoxification and liver protection.

## 1. Introduction

Alcohol has been classified as one of the human carcinogens by the International Agency for Research on Cancer. Alcoholic liver disease (ALD) is the leading cause of alcohol-related deaths, accounting for about 90% in some countries [1]. The liver is the main target organ for alcohol and toxic substances, and alcoholic liver injury caused by alcohol has become a major risk to human health. If alcoholic liver injury is left untreated for a long time, other organs of the organism will also be damaged [2]. Therefore, the search for effective and safe hepatoprotective substances is crucial for the prevention and treatment of alcoholic liver injury.

The repair of alcohol-induced liver injury is mainly achieved through the following three pathways. The first is reducing the absorption rate of alcohol in the gastrointestinal tract, reducing the amount of alcohol entering the bloodstream to reduce the burden on the liver. The second is regulating the activity of liver metabolic enzyme, alcohol dehydrogenase (ADH) [3], to accelerate alcohol metabolism, thereby reducing ethanol damage to the liver and brain, etc. The third is enhancing the organism’s ability to scavenge radicals, eliminating the excessive radicals generated by alcohol metabolism. This can effectively curb the damage caused by radicals to the liver and maintain the liver’s health. The most effective treatment for ALD is abstinence from alcohol, which is difficult to realize, and drug treatment has the limitations of side effects and individual differences in efficacy. Foreign hepatoprotective products for ALD are mainly used for symptomatic treat-ment, with fast efficacy. Such products have a certain effect on acute alcohol intoxication but have little effect on alcoholic liver injury [4]. Domestic hepatoprotective products are mainly used to inhibit the absorption of ethanol in the gastrointestinal tract, enhance the activity of ADH and aldehyde dehydrogenase (ALDH), thus promoting the high-speed metabolism of ethanol, reducing the content of acetaldehyde, and alleviate the damage of alcohol and its metabolites to the organism [5].

As reported, a high percentage of branched-chain amino acids (BCAA) can promote the expression of peroxidase-related genes, enhance the organism’s immunity, improve the regenerative function of hepatocytes, and effectively alleviate the deterioration of liver disease in patients [6]. Therefore, high Fischer ratio oligopeptides (HFOPs) with a high content of BCAA can be used for the treatment of liver injury. HFOPs are low molecular weight functional, active peptides characterized by a high content of BCAA and low content of aromatic amino acids (AAA), with a required BCAA to AAA molar ratio of more than twenty generally [7]. Due to the unique amino acid composition, HFOPs exhibit unique physiological activities in hepatoprotection, antioxidant, and treatment of phenylketonuria [8]. In recent years, there have been more and more reports on HFOPs, which have become one of the hotspots in the study of bioactive peptides. Chen et al. [9] obtained HFOPs from Antarctic krill by enzymatic hydrolysis with alcalase and flavourzyme. They evaluated their antioxidant activity in vitro, finding that Antarctic krill HFOPs exhibited high radical scavenging and reducing abilities and could react with peroxide radicals to delay lipid peroxidation, demonstrating good antioxidant capacity. Qin et al. [10] found through both in vivo and in vitro experiments that HFOPs extracted from goat whey had a strong ability to scavenge radicals and could reduce the content of malondialdehyde (MDA) caused by oxidative stress and increase the activity of superoxide dismutase (SOD) and glutathione-peroxidase (GSH-Px).

*Pueraria lobata* (PL) is a medicinal and edible plant with the same origin [11], which has a variety of pharmacological properties [12]. PL is a popular raw material for hepatoprotective quality against alcohol-induced injury. Zhao et al. [13] investigated the effect of probiotic fermentation treatment of PL on alcoholic liver injury in rats through in vivo experiments, finding that fermented PL extract reduced alcohol-induced liver enlargement, increased antioxidant capacity, reduced inflammation levels, and was able to effectively alleviate liver injury, achieving hepatoprotective effects. Therefore, PL can be added to healthy foods as a natural active ingredient with great market potential.

The HFOPs prepared in our laboratory showed good effects of hepatoprotection against alcohol-induced injury. In this study, HFOPs were compounded with PL at a ratio of 1:1 (*w*/*w*), abbreviated as HFOPs + PL(1:1), and the anti-alcoholic and hepatoprotective effect of the HFOPs with/without half substitution by PL were compared, to evaluate the anti-alcoholic and hepatoprotective effect of HFOPs + PL(1:1). Firstly, the anti-alcoholic and antioxidant effects of HFOPs + PL(1:1) were investigated by analyzing and evaluating ADH and SOD activities through in vitro chemical methods. In vitro chemical methods are fast, simple, and low cost. However, they cannot reflect the mechanism of hepatoprotective substances in the organism. At the same time, HepG2 cells have similar metabolic pathways to normal human hepatocytes and are widely used as cell models to study alcoholic liver injury [14]. Thus, HepG2 cells were further selected as model cells to simulate alcohol injury, and an alcohol-injured HepG2 cell model was constructed. The protective effects of HFOPs + PL(1:1) on HepG2 cells and HepG2 injured cells were evaluated by CCK-8 assay for cell survival rate. Then, the release of alanine aminotransferase (ALT) and aspartate aminotransferase (AST), the activities of antioxidant enzymes SOD and GSH, the content of lipid peroxidation products MDA, and the content of cellular inflammatory factors tumor necrosis factor-α (TNF-α) and interleukin-6 (IL-6) were determined to clarify the protective effect of HFOPs + PL(1:1) on alcohol-injured HepG2 cells. Then, an acute alcoholic liver injury model was constructed using Balb/C mice as test subjects. The changes in AST and ALT levels in serum and ADH, ALDH, SOD, GSH-Px, CAT, and MDA contents in liver tissues were detected so as to systematically evaluate the protective effects of HFOPs + PL(1:1) on mice with acute alcoholic liver injury. This study will provide a theoretical basis for developing detoxification and hepatoprotection products.

## 2. Materials and Methods

### 2.1. Materials and Reagents

The processing by-products of scallops (*Chlamys farreri*), which remove the shells and scallop columns, were purchased from Yantai Hongli Aquatic Market, Shandong Province, China. PL extract was purchased from Xi’an Huilin Biotechnology Co. (Xi’an, China). Human hepatic HepG2 cells were obtained from the Cell Bank of the Typical Culture Preservation Committee of the Chinese Academy of Sciences (Wuhan, China). Healthy male Balb/c mice (SPF grade), weighing (20 ± 2) g, were purchased from Jinan Pengyue Experimental Animal Breeding Co., Ltd. (Jinan, China), with Animal Production License No. of SCXK (Lu) 20190003. Additionally, 56° baijiu was purchased from Beijing Red Star Co., Ltd. (Beijing, China). SOD, and oxidized coenzyme I was purchased from Shanghai Yuanye Biotechnology Co. (Shanghai, China). ADH was purchased from Beijing Solebo Technology Co. (Beijing, China). Furthermore, 1640 culture medium, trypsin digestion solution, penicillin-streptomycin solution (double antibody), and fetal bovine serum (FBS) were purchased from Wuhan Pnosay Life Technology Co. (Wuhan, China). Cell Counting Kit-8 was purchased from Shanghai Biyuntian Biotechnology Co. (Shanghai, China). Alanine Aminotransferase (ALT/GPT) Activity Assay Kit, Aspartate Aminotransferase (AST/GOT) Activity Assay Kit, BCA Protein Assay Kit, MDA Assay Kit, SOD Assay Kit, Micro Reduced Glutathione (GSH) Assay Kit, ADH Assay Kit, ALDH Assay Kit, GSH-Px Assay Kit, human TNF-α ELISA Kit, Catalase (CAT) Assay Kit, total protein (TP) quantitative assay kit, and human IL-6 ELISA kit were purchased from Nanjing Jiancheng Bioengineering Institute (Nanjing, China).

All other chemical reagents were of analytical grade.

### 2.2. Preparation of HFOPs

The processing by-products of scallops were homogenized with 5× weight of deionized water. The homogenate was hydrolyzed by pepsin and flavourzyme. The enzyme activity was set at 1000 U/g. Reaction T and pH were set at the optimum of each protease (pepsin at pH 2.5, 37.0 °C and flavourzyme at pH 7.0, 50 °C). The reaction time was 2 h. The hydrolysis was terminated in boiling water for 15 min. The hydrolysate was centrifuged at 10,000 g for 10 min, and the supernatant was collected and mixed with activated carbons at pH 7.0, 25 °C, with a solid/liquid ratio (*w*/*v*) of 1:15 g/mL for 1 h. The hydrolysate was subjected to ultrafiltration with a 0.45 μm microporous filter membrane to clear away the adsorbents. Then, 3 kDa and 200 Da ultrafiltration membranes were used to remove peptides with high molecular weights and salt in the hydrolysate [15]. The fraction with a molecular weight segment of 200~3000 Da, defined as HFOPs, was collected, reduced pressure distilled, and freeze-dried for further experiment.

### 2.3. Evaluation of In Vitro Activity of HFOPs, PL, and HFOPs + PL(1:1) on Hepatoprotective Against Alcohol-Induced Injury and Hepatoprotection

#### 2.3.1. Preparation of HFOPs, PL, and HFOPs + PL(1:1) Solutions

HFOPs, PL solution: PL and HFOPs were prepared as 1~5 mg/mL solution, respectively, centrifuged, and filtered through 0.45 μm filter membrane to be measured.

HFOPs + PL(1:1) solution: HFOPs and PL with the same weight were mixed together and prepared a solution with a total concentration of 5 mg/mL.

#### 2.3.2. Determination of ADH Activity

The in vitro ADH activity was determined by the method of Wang et al. [16] with some modifications. Sodium pyrophosphate buffer (pH 8.8), nicotinamide adenine dinucleotide (NAD+), ethanol, and the sample were added into the test tube in sequence and mixed well. Distilled water was used as the control. The mixture was heated in a 25 °C water bath for 5 min, immediately taken out and added 0.1 mL of ADH solution, and mixed well. The absorbance value at 340 nm wavelength was detected, with 30 s reading intervals for 5 min consecutively.

#### 2.3.3. Determination of SOD Activity

SOD activity was determined by referring to the method of Wang et al. [17] with slight modification. The pyrogallol autoxidation rate was determined and controlled at 0.07 (±0.002) OD/min. Tris-HCl buffer, the sample, and the SOD solution were mixed and placed in a water bath at 25 °C for 10 min. Then, pyrogallol solution was added. After thorough mixing, the detection was carried out at 325 nm wavelength with 30 s reading intervals for 3 min consecutively.

#### 2.3.4. Determination of the Scavenging Capacity of Hydroxyl Radicals, DPPH Radicals, and ABTS Radicals

The hydroxyl radical activity was determined by referring to the method of Li et al. [18].

The DPPH radical activity was determined according to the method of Zheng et al. [19]. An amount of 50 μL of the sample was mixed with an equal volume of DPPH solution (0.1 mmol/L) in a 96-well plate, then incubated for 30 min in the dark for 30 min, and then measured at 517 nm.

The ABTS radical activity was determined referring to the method of Xiao Hu et al. [20], with ultrapure water as the blank and 50% methanol as the control.

### 2.4. Protective Effect of HFOPs, PL, and HFOPs + PL(1:1) on Alcohol-Injured HepG2 Cells

#### 2.4.1. HepG2 Cell Viability

After diluting the cell concentration of 2.5 × 10^5^/mL, 100 μL/well was inoculated into a 96-well cell culture plate and cultured for 24 h. HFOPs and PL were prepared with culture medium at concentrations of 10 μg/mL, 20 μg/mL, 30 μg/mL, 40 μg/mL, 50 μg/mL, 60 μg/mL, and 70 μg/mL, respectively, and added into HepG2 cells. After 24 h, 48 h, and 72 h of incubation, the supernatant was removed, and the culture medium containing 10% CCK-8 was added, incubating for 1 h in the dark. The absorbance value of each well was measured at 450 nm, with culture medium as the control. Six parallels were set in each group, and the survival rate of HepG2 cells was calculated to analyze whether HFOPs and PL had toxic effects on HepG2 cells within this concentration range.

#### 2.4.2. Alcohol-Injured HepG2 Cell Viability

After 24 h of cultivation in the incubator, the supernatant was removed. The experimental groups were added with culture medium containing HFOPs (10 μg/mL, 20 μg/mL, 30 μg/mL, 40 μg/mL, 50 μg/mL, 60 μg/mL, and 70 μg/mL), PL (10 μg/mL, 20 μg/mL, 30 μg/mL, 40 μg/mL, 50 μg/mL, 60 μg/mL, and 70 μg/mL), and HFOPs + PL (1:1, 50 μg/mL), respectively, and ethanol with a determined final alcohol concentration of 0.6 mol/L; the normal group was replaced with culture medium. The model group was replaced with a culture medium with 0.6 mol/L ethanol. The incubation continued for 24 h, and the supernatant was removed. A culture medium containing 10% CCK-8 was added, and the cells were incubated in the dark for 1 h. The absorbance value of each well was determined at 450 nm, with culture medium as the control. Six parallels were set in each group, and the survival rate of HepG2 cells was calculated.

#### 2.4.3. Effects of HFOPs, PL, and HFOPs + PL(1:1) on Liver Function Indicators, Antioxidant Activity, and Inflammatory Factors in Alcohol-Injured HepG2 Cells

HepG2 cells in the logarithmic growth stage were taken and resuspended as a cell suspension. After counting, the cell density was adjusted to 5 × 10^4^/mL, and the cells were plated to 6 well plates with 2 mL/well. After 24 h of cultivation in the incubator, the supernatant was removed, and GSH was selected as the positive control group. The experimental sample group was added with a culture medium containing GSH 50 μg/mL, HFOPs 50 μg/mL, PL50 μg/mL, and HFOPs + PL(1:1) 50 μg/mL, and ethanol with a final concentration of 0.6 mol/L. The normal group was treated with a different culture medium, while the model group was replaced with a culture medium with 0.6 mol/L ethanol. After cultivation, the supernatant and cells of each group were collected for relevant activity determination.

The requirements of the reagent kit instructions were strictly followed to determine AST and ALT, SOD and GSH activity, MDA content, and the release of inflammatory factors TNF-α and IL-6 in cell culture medium.

### 2.5. In Vivo Experiment in Mice

#### 2.5.1. Grouping and Model Establishment of Animal Experiment

Preliminary experiments were conducted to compare different HFOPs-to-PL ratios (1:1, 1:2, and 1:3). Based on the results, the ratio of 1:1 was identified as optimal and was therefore chosen for further investigation. The acute alcoholic liver injury model was established by referring to the related literature [21] with some modifications. Male Balb/c mice were housed in an environment with a temperature of 20 ± 2 °C, humidity of 50~60%, and alternating darkness and light. After 3 days of adaptive feeding, the mice were randomly divided into 8 groups (8 mice in each group). The positive control group was gavaged 50 mg·kg^−1^ of GSH, the HFOPs group was gavaged 400 mg·kg^−1^ of HFOPs, the PL group was gavaged 400 mg·kg^−1^ of PL, the low, medium, and high-dose groups of HFOPs + PL(1:1), named HFOPs + PL(1:1)-L, HFOPs + PL(1:1)-M, HFOPs + PL(1:1)-H, were gavaged with a total administered dose of 400 mg·kg^−1^, 600 mg·kg^−1^, and 800 mg·kg^−1^ of HFOPs + PL(1:1), respectively, and the normal and model group were gavaged with an equal amount of distilled water for 4 days. After 1 h of the last gavage, each group except the normal group was gavaged with 56° baijiu according to the determined optimal gavage dosage of 0.9 mL·10g^−1^. The normal group was gavaged with distilled water of the same volume. The drunkenness time and sobriety time of each group of mice were recorded. After 24 h, all mice were sacrificed after taking blood from the orbits, the serum was centrifuged, and the liver was quickly dissected and removed, weighed and recorded, and then stored in a refrigerator at −80 °C.

#### 2.5.2. Determination of Drunkenness and Sobriety Time in Mice

The behavior of mice after gavage alcohol was observed, and the drunkenness and sobriety time were recorded. When mice were in the supine state, three self-corrections within each minute were used as the criterion for the recovery of the flip-flop reflex [22]. Drunkenness time refers to the time period from the infusion of alcohol to the disappearance of the flip reflex, and sobriety time is the time period from the disappearance of the flip reflex to the time when the mice can self-correct to restore the flip reflex.

#### 2.5.3. Determination of Liver Index

The liver index was calculated according to the method described by Li [23] et al.

(1) Liver Index=Liver weight(g)/mouse weight(g)×100%

#### 2.5.4. Determination of Serum Indexes

The blood was collected from mice by removing the eyeballs and placed in a centrifuge tube for natural coagulation, then centrifuged at 4 °C for 10 min (3000 rpm) to separate the serum, which was stored in a refrigerator at −80 °C. ALT and AST activities were determined [24] according to the requirements of the assay kit.

#### 2.5.5. Determination of Biochemical Parameters in Liver Tissue

Liver tissue was homogenized and centrifuged at 3000 rpm at 4 °C for 15 min. The protein content, ADH, ALDH, CAT, GSH-Px, SOD activity, and MDA content of liver tissue were determined [25] according to the requirements of the assay kit.

### 2.6. Statistical Analysis

All values were shown as the means of three different tests with duplicate treatments per experiment. Microsoft Excel 2016 software was used to analyze the average of single-factor experimental data. Statistical analysis was performed using variance analysis (ANOVA). The results were expressed as mean ± SD, and the differences were considered statistically significant at *p* < 0.05.

## 3. Results

### 3.1. Effects of HFOPs, PL, and HFOPs+PL(1:1) on ADH and Antioxidant Activities In Vitro

**Figure 1:**
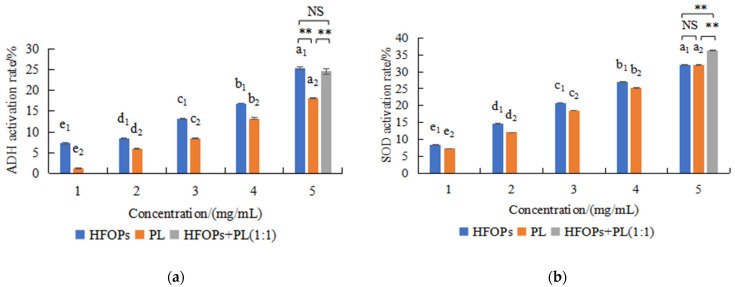
Effects of HFOPs, PL, and HFOPs + PL(1:1) on ADH (**a**) and SOD (**b**) activities in vitro. Different lowercase letters above the bars indicate significant differences (*p* < 0.05). Different numerical labels represent distinct significance groups. **: *p* < 0.05, NS: no statistically significant difference.

The effects of HFOPs and PL on ADH and SOD activities were investigated (Figure 1), finding that within the concentration range of 1~5 mg/mL, both HFOPs and PL had a significant promotion effect on ADH activity and SOD activity, becoming more and more significant with the increase in concentration. Both of them showed a gradient enhancement effect with a dose-effect relationship. Then, the ADH activity and SOD activity of HFOPs + PL(1:1) with a total concentration of 5 mg/mL was determined. It was found that the HFOPs + PL (1:1) mixture, in which half of the HFOPs were replaced by PL, showed no significant difference in antioxidant activity compared to HFOPs alone, but was significantly higher than that of PL (*p* < 0.05). Furthermore, this mixture significantly enhanced superoxide dismutase (SOD) activity, with an effect substantially higher than that of either HFOPs or PL alone (*p* < 0.05).

**Figure 2:**
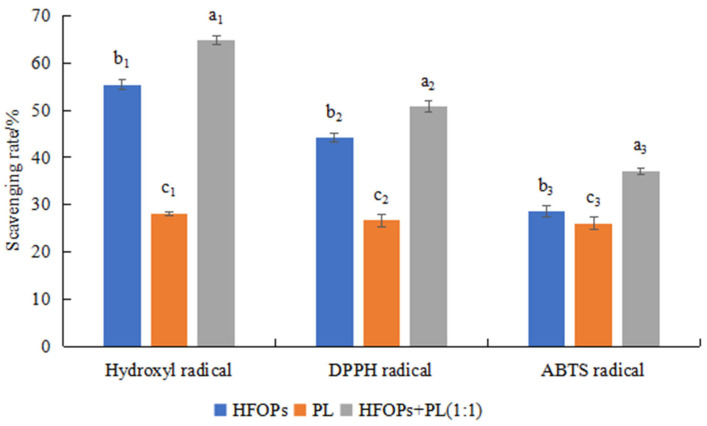
Effects of HFOPs, PL, and HFOPs + PL(1:1) on the scavenging rate of hydroxyl radicals, DPPH radicals, and ABTS radicals. Different lowercase letters above the bars indicate significant differences (*p* < 0.05). Different numerical labels represent distinct significance groups.

The antioxidant effects of HFOPs, PL, and HFOPs + PL(1:1) were further investigated. Figure 2 showed the scavenging rate of HFOPs, PL, and HFOPs + PL(1:1) on hydroxyl radicals, DPPH radicals, and ABTS radicals. It was found that the scavenging ability of HFOPs + PL(1:1) for the three radicals was significantly higher than that of both HFOPs and PL (*p* < 0.05). It was hypothesized that HFOPs + PL(1:1) with half substitution of HFOPs by PL might have better antioxidant activity than HFOPs and PL. The effects of HFOPs + PL(1:1) on hepatoprotection against alcohol-induced injury and hepatoprotection were further investigated by cell and mouse experiments.

### 3.2. Protective Effects of HFOPs, PL, and HFOPs + PL(1:1) on Alcohol-Injured HepG2 Cells

#### 3.2.1. Effects of HFOPs and PL on HepG2 Cell Viability

**Figure 3:**
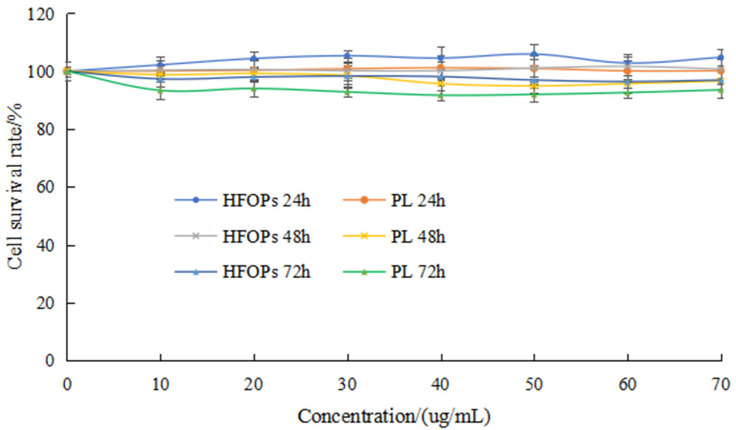
Effects of HFOPs and PL with different concentrations and different action times on the survival rate of HepG2 cells.

Figure 3 showed the effects of HFOPs and PL with different concentrations (10 μg/mL, 20 μg/mL, 30 μg/mL, 40 μg/mL, 50 μg/mL, 60 μg/mL, and 70 μg/mL) and different action time (24 h, 48 h, and 72 h) on the survival rate of HepG2 cells, finding that compared with the normal group, HFOPs and PL at different concentrations had no significant effect on the survival rate of HepG2 cells under the same action time, and with action time changing, the cell survival rate of each group remained above 90%, indicating that HFOPs and PL had no toxic effects on the HepG2 cells in the concentration range of 10~70 μg/mL. In addition, the cell survival rate at 24 h was slightly higher than that at 48 h and 72 h. Therefore, the optimal action time for HFOPs and PL was determined to be 24 h within the concentration range of 10~70 μg/mL.

#### 3.2.2. Effects of HFOPs, PL, and HFOPs + PL(1:1) on Alcohol-Injured HepG2 Cell Viability

**Figure 4:**
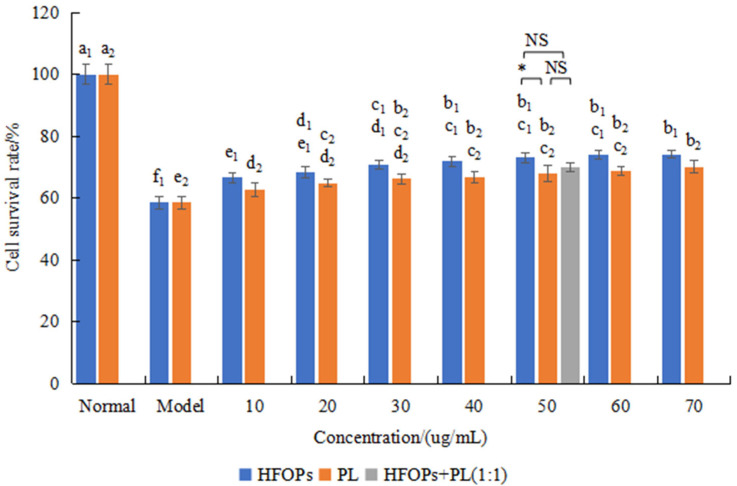
Alcohol-injured HepG2 cell viability. Different lowercase letters above the bars indicate significant differences (*p* < 0.05). Different numerical labels represent distinct significance groups. *: *p* < 0.05, NS: no statistically significant difference.

Figure 4 showed the effects of HFOPs, PL, and HFOPs + PL(1:1) on the survival rate of alcohol-injured HepG2 cells. It was found that the cell survival rate of the model group was 58.5 ± 1.92%, significantly lower than that of the normal group (*p* < 0.05), indicating the successful construction of the alcohol-injured HepG2 cell model. Compared with the model group, the cell survival rate of the sample group with different concentrations of HFOPs and PL significantly increased and showed an upward trend with the increase in concentration (*p* < 0.05), indicating that HFOPs and PL had a protective effect on alcohol-injured HepG2 cells within the concentration range.

The effect of HFOPs + PL(1:1) on the survival rate of alcohol-injured HepG2 cells was further investigated. The survival rate of HFOPs + PL(1:1) with a total concentration of 50 μg/mL was compared with that of the HFOPs and PL at the same concentration. It was found that the cell survival rate of HFOPs + PL(1:1) was not significantly different from that of both HFOPs and PL and had a protective effect on alcohol-injured HepG2 cells.

#### 3.2.3. Effects of HFOPs, PL, and HFOPs + PL(1:1) on the AST and ALT in Alcohol-Injured HepG2 Cells

**Figure 5:**
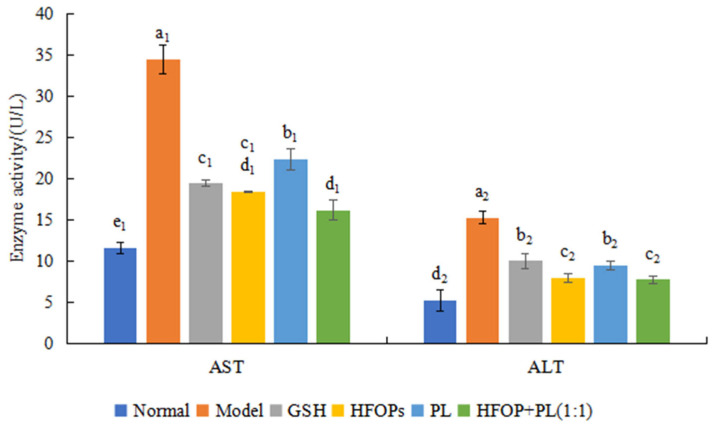
The AST and ALT activities in alcohol-injured HepG2 cells. Different lowercase letters above the bars indicate significant differences (*p* < 0.05). Different numerical labels represent distinct significance groups.

The AST and ALT levels of the cells in each group were shown in Figure 5. Compared with the normal group, the model group showed a significant increase in AST and ALT activity after alcohol treatment on HepG2 cells, reaching the highest values of 32.52 ± 1.75 U/L and 15.33 ± 0.70 U/L, respectively. After being treated with HFOPs, PL, and HFOPs + PL(1:1), the AST and ALT activities of HepG2 injured cells were all reduced, being significantly lower than that of the model group (*p* < 0.05). HFOPs + PL(1:1) showed no significant difference in AST activity and lower ALT activity compared with HFOPs, which was significantly lower than that of PL and the positive control GSH (*p* < 0.05). The above results indicated that all sample groups could reduce the leakage of AST and ALT in alcohol-injured HepG2 cells and had a protective effect on the alcoholic injury of HepG2 cells. HFOPs + PL(1:1) with half substitution of HFOPs by PL had the best protective effect, which was significantly higher than that of the positive control GSH (*p* < 0.05).

#### 3.2.4. Effects of HFOPs, PL, and HFOPs + PL(1:1) on the Antioxidant Activity in Alcohol-Injured HepG2 Cells

**Figure 6:**
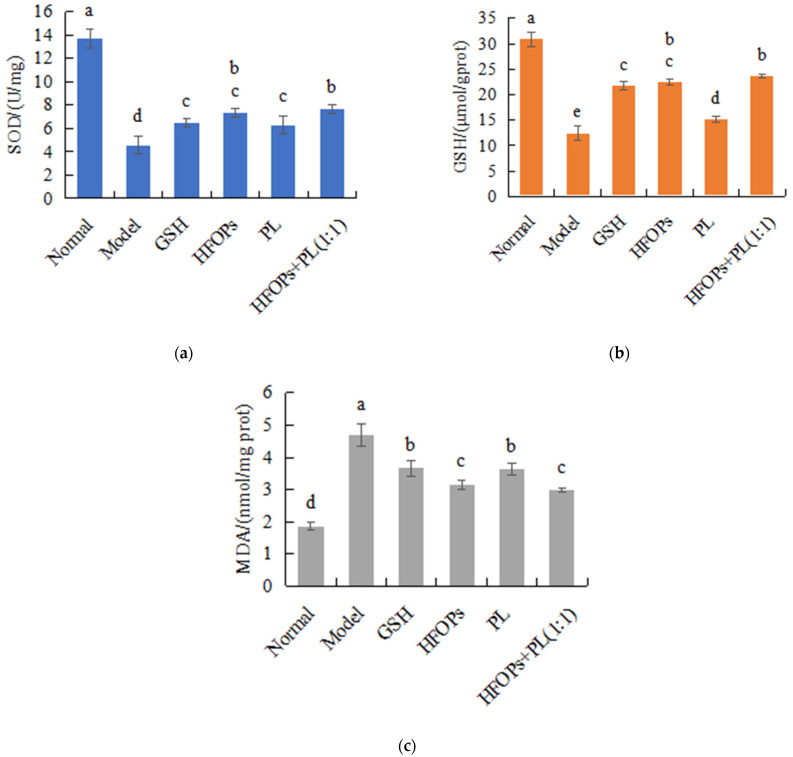
The SOD (**a**) activity, GSH (**b**) activity, and MDA (**c**) content in alcohol-injured HepG2 cells. Different lowercase letters above the bars indicate significant differences (*p* < 0.05).

In this cellular experimental design, GSH serves as the positive control. The addition of exogenous GSH is intended to elevate GSH levels exclusively in the positive control group without affecting the GSH levels in other treatment groups. Figure 6 showed the effects of HFOPs, PL, and HFOPs + PL(1:1) on the SOD activity, GSH activity, and MDA content in alcohol-injured HepG2 cells. It was found that, compared with the normal group, the intracellular SOD and GSH activities of the model group were significantly reduced (*p* < 0.05), and the enzyme activities of all sample groups were significantly higher than those of the model group (*p* < 0.05). Moreover, HFOPs + PL(1:1) had the same enzyme activities as HFOPs, significantly higher than PL and the positive control GSH (*p* < 0.05).

As shown in Figure 6, compared with the normal group, the model group showed a significant increase in MDA content (*p* < 0.05), and the MDA content of all sample groups was significantly lower than those of the model group (*p* < 0.05). Moreover, HFOPs + PL(1:1) had the same MDA content as HFOPs, significantly lower than that of PL and the positive control GSH (*p* < 0.05). The result was consistent with the antioxidant enzyme results described above, indicating that HFOPs, PL, and HFOPs + PL(1:1) inhibited alcohol-induced oxidative stress by increasing antioxidant enzyme activity and reducing the generation pathways of lipid peroxidation product, thereby reducing alcohol-induced HepG2 cell injury. The antioxidant stress effect of HFOPs + PL(1:1) with half substitution of HFOPs by PL remained unchanged compared with HFOPs, significantly better than that of the positive control GSH (*p* < 0.05).

#### 3.2.5. Effects of HFOPs, PL, and HFOPs + PL(1:1) on TNF-α and IL-6 Content in Alcohol-Injured HepG2 Cells

**Figure 7:**
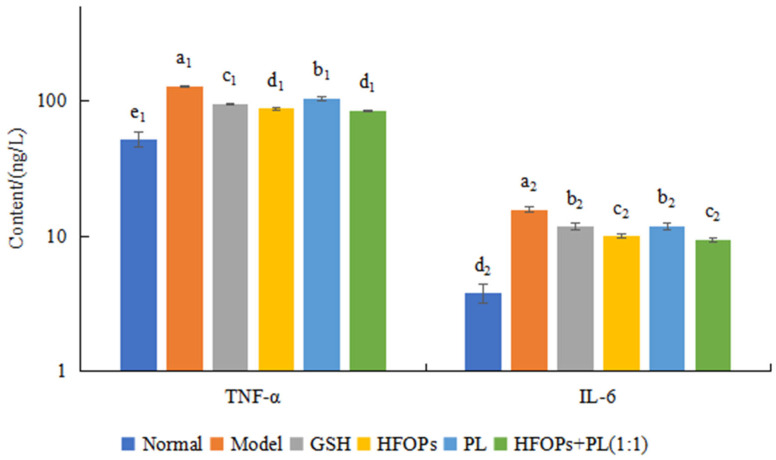
The TNF-α and IL-6 content in alcohol-injured HepG2 cells. Different lowercase letters above the bars indicate significant differences (*p* < 0.05). Different numerical labels represent distinct significance groups.

The cellular inflammation level was evaluated by determining the content of TNF-α and IL-6 in the cell culture medium, and the results were shown in Figure 7. Ethanol acted on cells and stimulated the secretion of TNF-α and IL-6. After treating injured cells with HFOPs, PL, and HFOPs + PL(1:1), the release of TNF-α and IL-6 in the cell culture medium was significantly reduced (*p* < 0.05), indicating that all of them could effectively reduce the release of inflammatory factors TNF-α and IL-6 from HepG2-injured cells, thereby inhibiting cellular inflammatory responses and demonstrating potential anti-inflammatory effects. HFOPs + PL(1:1) showed no significant difference in effect from HFOPs, while being significantly better than PL (*p* < 0.05), indicating that the mixture with half substitution of HFOPs by PL maintained anti-inflammatory effects comparable to HFOPs.

### 3.3. Protective Effect of HFOPs, PL, and HFOPs+PL(1:1) on Acute Alcoholic Liver Injury in Mice

#### 3.3.1. Effects of HFOPs, PL, and HFOPs + PL(1:1) on the Drunkenness Time, Sobriety Time, and Liver in Drunken Mice

In the animal experimental design, the HFOPs-alone and PL-alone groups were administered doses of 400 mg·kg^−1^, respectively. In the HFOPs + PL(1:1)-L group, HFOPs and PL were each administered at a dose of 200 mg·kg^−1^, while in the HFOPs + PL(1:1)-M group, HFOPs and PL were each administered at a dose of 400 mg·kg^−1^. This design, incorporating half-doses of HFOPs and PL in the combination group, not only facilitates the comparison of the anti-alcoholic and hepatoprotective effects among the HFOPs-alone, PL-alone, and HFOPs + PL(1:1)-L groups under equivalent total administration conditions, but also, through the inclusion of the HFOPs + PL(1:1)-M group alongside the HFOPs-alone and PL-alone groups, allows for further investigation into the alcohol-induced hepatoprotective effects of the full-dose combination compared to the individual components. The behavior of mice after gavage of alcohol was observed, and the drunkenness and sobriety time of mice was recorded, with the results shown in Table 1.

Compared with the model group, the drunkenness time was prolonged in all sample groups. At the same total administered dose, HFOPs + PL(1:1)-L had the same drunkenness time with HFOPs, higher than that of PL and the positive control GSH. The drunkenness time of HFOPs + PL(1:1) tended to be prolonged with the increase in the dose in a dose-dependent relationship.

Compared with the model group, the sobriety time of mice in all sample groups was reduced. At the same total administered dose, the sobriety time of HFOPs + PL(1:1)-L was not significantly different from that of HFOPs, lower than that of PL and the positive control GSH. The sobriety time was shortened by HFOPs + PL(1:1) with increasing dose, indicating a dose-dependent effect.

Compared with the normal group, the liver index of mice in the model group was significantly increased (*p* < 0.05), indicating that alcohol intake caused liver damage and swelling in mice, leading to an increase in liver index. Compared with the model group, the liver index was significantly reduced (*p* < 0.05). At the same total administered dose, the liver index of HFOPs + PL(1:1)-L had no significant difference with that of HFOPs and PL.

#### 3.3.2. Effects of HFOPs, PL, and HFOPs + PL(1:1) on ADH and ALDH in the Liver of Mice with Acute Alcoholic Liver Injury

**Figure 8:**
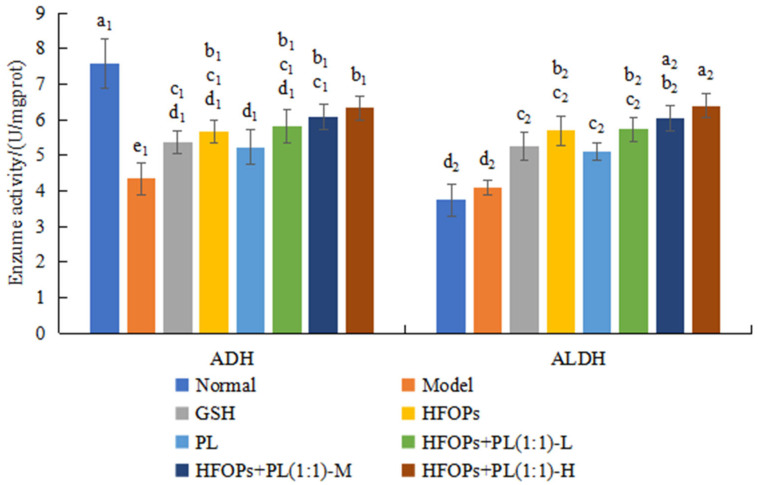
The ADH and ALDH in the liver of mice with acute alcoholic liver injury. Different lowercase letters above the bars indicate significant differences (*p* < 0.05). Different numerical labels represent distinct significance groups.

The ADH and ALDH activities were used to evaluate the rate of alcohol metabolism in the liver, and the results were shown in Figure 8. Compared with the normal group, the ADH level significantly decreased (*p* < 0.05), and the ALDH level increased in the liver tissues of mice in the model group. Compared with the model group, ADH and ALDH levels were significantly improved in the livers of mice in all sample groups (*p* < 0.05). At the same total administered dose, ADH and ALDH activities of HFOPs+PL(1:1)-L were not significantly different from those of HFOPs. The levels of ADH and ALDH in the HFOPs + PL(1:1)-M group were not significantly different from those in the HFOPs group but were significantly higher than in the PL group (*p* < 0.05). The ADH and ALDH activities of HFOPs + PL(1:1) were dose-dependent with the increasing dose.

#### 3.3.3. Effects of HFOPs, PL, and HFOPs + PL(1:1) on Serum ALT and AST in Mice with Acute Alcoholic Liver Injury

**Figure 9:**
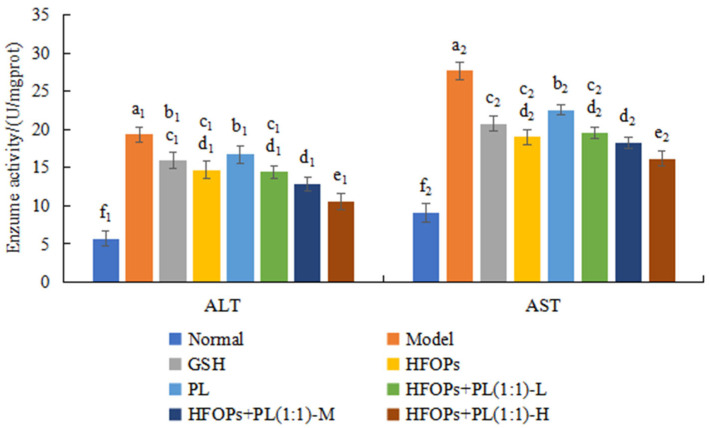
The serum ALT and AST in mice with acute alcoholic liver injury. Different lowercase letters above the bars indicate significant differences (*p* < 0.05). Different numerical labels represent distinct significance groups.

The serum levels of ALT and AST are elevated, and the results were shown in Figure 9. Compared with the normal group, the serum ALT and AST activities of mice in the model group were significantly increased (*p* < 0.05), indicating the successful construction of the model of acute alcoholic liver injury in mice. Compared with the model group, the serum ALT and AST activities of all sample groups were significantly decreased (*p* < 0.05). At the same total administered dose, the serum ALT and AST activities of HFOPs + PL(1:1)-L were not significantly different from those of HFOPs, and were significantly lower than those of PL (*p* < 0.05). The effect of the HFOPs + PL(1:1)-M group was comparable to that of the HFOPs + PL(1:1)-L group. However, the HFOPs + PL(1:1)-H group exhibited a significantly greater effect than both the HFOPs + PL(1:1)-M and HFOPs + PL(1:1)-L groups (*p* < 0.05). The ALT and AST activities of HFOPs + PL(1:1) were dose-dependent with the increasing dose.

#### 3.3.4. Effects of HFOPs, PL, and HFOPs + PL(1:1) on Antioxidant Enzyme Activity and MDA Content in the Liver of Mice with Acute Alcoholic Liver Injury

**Figure 10:**
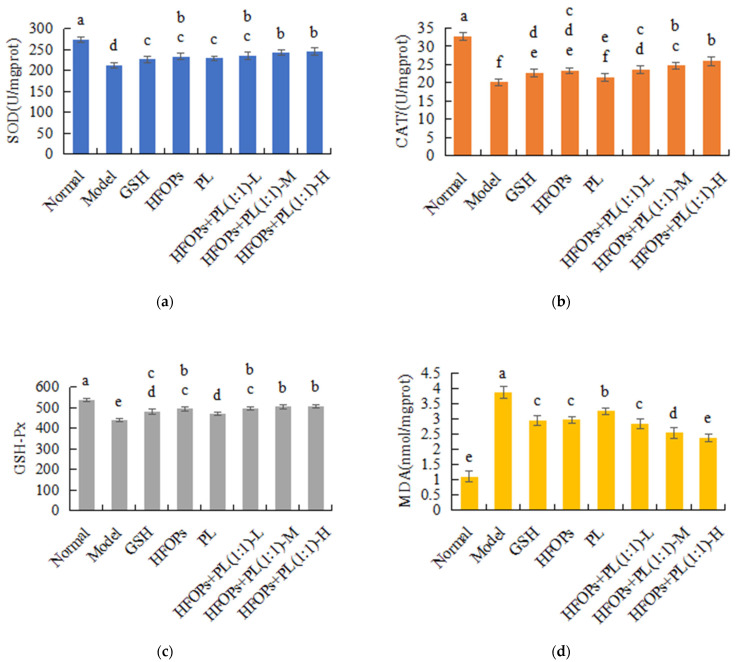
The SOD (**a**), CAT (**b**), GSH-Px (**c**) activities, and MDA (**d**) content in the liver of mice with acute alcoholic liver injury. Different lowercase letters above the bars indicate significant differences (*p* < 0.05).

Figure 10 showed the effects of HFOPs, PL, and HFOPs + PL(1:1) on the SOD, CAT, GSH-Px activities, and MDA content in the liver of mice with acute alcoholic liver injury. It was found that compared with the normal group, after gavage alcohol, the GSH-Px, SOD, and CAT activities were significantly decreased (*p* < 0.05), and the MDA content was significantly increased in the livers of mice in the model group (*p* < 0.05), so the antioxidant protection system was damaged, leading to oxidative stress and liver injury in mice. Compared with the model group, the levels of CAT, SOD, and GSH-Px in all sample groups were significantly increased (*p* < 0.05), while the MDA content was significantly decreased (*p* < 0.05). At the same total administered dose, the CAT, SOD, and GSH-Px activities and the MDA content were not significantly different from those of HFOPs (*p* < 0.05), and were significantly higher than those of PL (*p* < 0.05). The HFOPs + PL(1:1)-M group exhibited significantly lower MDA content compared to the HFOPs, PL, and GSH groups (*p* < 0.05). Furthermore, its CAT, SOD, and GSH-Px levels were significantly higher than those in the PL group but showed no significant difference from the HFOPs group (*p* < 0.05). The changes in CAT, SOD, and GSH-Px activities and MDA content of HFOPs + PL(1:1) were dose-dependent with the increasing dose.

## 4. Discussion

More than 90% of the alcohol ingested by the organism is metabolized in the liver, converted into acetaldehyde by ADH, and then metabolized into non-toxic acetic acid by ALDH [26]. So, one of the main ways to improve alcohol-induced liver injury and protect liver is to regulate the activity of the liver metabolic enzymes, ADH and ALDH, to accelerate alcohol metabolism [5], which converts alcohol into CO_2_ and H_2_O through a series of biochemical reactions and excretes them from the body. Both in vitro chemical experiments and in vivo mouse experiments have shown that HFOPs + PL(1:1) with half substitution of HFOPs by PL had the same effect to alcohol-induced liver injury with HFOPs, and could significantly increase the activities of ethanol-related metabolic enzymes, ADH and ALDH, thereby accelerating the breakdown metabolism of alcohol in the liver and reducing the risk of liver injury caused by alcohol and its harmful metabolite acetaldehyde.

ALT and AST are enzymes that typically exist in liver cells. ALT and AST activities are commonly used indicators for detecting liver injury, and ALT and AST levels are the most sensitive biomarkers for detecting liver cell damage [27]. ALT is located in the nucleus of liver cells, while AST is located in mitochondria. Pathogenic factors cause liver cell deformation and changes in cell membrane permeability, which in turn leads to the leakage of ALT from liver cells, increasing serum ALT levels [28]. When liver cell damage progresses, mitochondria are affected, leading to an increase in serum AST levels. ALT and AST in serum are at low levels under normal conditions. When the liver fails to break down alcohol in time, ALT and AST in hepatocytes leak out of the cells into the bloodstream after liver injury [29], causing damage to the cell membrane and mitochondrial structure and resulting in changes in permeability. In vitro cell experiments and in vivo mouse experiments have shown that HFOPs + PL(1:1) with half substitution of HFOPs by PL had the same hepatoprotective effect with HFOPs and could alleviate the damage to hepatocytes and mitochondria caused by alcohol intake, protect the permeability and integrity of the liver cell membranes of mice, and reduce the leakage of ALT and AST. Yu et al. [30]. reported that for mice with alcohol-induced liver injury, the significant increase in serum ALT and AST activity was closely related to alcohol-induced liver injury, which was similar to our findings.

Oxidative stress is an important indicator reflecting the degree of liver injury, and alcohol metabolism can increase the levels of radicals and reactive oxygen species (ROS); excessive ROS can lead to oxidative stress, and GSH has the ability to clear ROS and is often used as a biomarker [31] to evaluate the degree of damage caused by oxidative stress. The activities of SOD, CAT, and GSH-Px can also reflect the degree of oxidative stress in the liver [32]. Increasing the activity of these enzymes can accelerate the liver’s ability to scavenge oxygen-free radicals, thereby protecting the liver. When the content of oxygen-free radicals exceeds the antioxidant clearance capacity of GSH, etc., lipid peroxidation occurs in the liver cell membrane, causing oxidative damage to the liver cell membrane, which can result in an increase in lipid peroxidation products such as MDA, leading to severe liver toxicity. MDA is not only one of the end products of membrane lipid peroxidation [33] but also an important indicator of oxidative stress occurrence [34]. The elevation of antioxidant enzyme activity can inhibit the increase in MDA content, thereby playing an antioxidant role. So, to enhance the organism’s ability to scavenge radicals, eliminating the excessive radicals generated by alcohol metabolism was also an important way to improve alcohol-induced liver injury and protect liver. Both in vitro cell experiments and in vivo mouse experiments have shown that HFOPs + PL(1:1) kept the same antioxidant capacity as HFOPs and could reduce alcohol-induced oxidative stress by regulating antioxidant enzyme activity and inhibiting lipid peroxidation levels in the liver of acute alcohol-poisoned mice, reducing the production of MDA in liver membrane lipids, effectively alleviating alcohol damage to the liver. It was reported that HFOPs in corn could effectively repair alcohol-induced liver injury in mice by improving serum biochemical indicators of liver function [35]. Zhao et al. [36] found that HFOPs prepared by enzymatic and fermentation methods increased blood lipids in mice, improved lipid metabolism disorders, reduced oxidative stress, and alleviated alcoholic liver injury. Qin et al. (2022) [10] reported that HFOPs in sheep whey have antioxidant capacity both in vivo and in vitro. Zhang et al. successfully prepared antioxidant peptides from walnut residue, which through both in vitro and in vivo experiments were found to significantly improve cellular damage caused by oxidative stress by regulating the levels of ROS, CAT, SOD, and GSH-Px [37].

Many studies have found that alcohol can induce the expression of cellular inflammatory factors, leading to the occurrence of inflammatory reactions [38]. After drinking alcohol, ethanol in the body is broken down into acetaldehyde, which interacts with cellular macromolecules, activating pro-inflammatory cytokines, interleukins, etc., thereby triggering an inflammatory response in the liver [39]. TNF-α and IL-6 are common inflammatory factors in hepatocellular injury [40]. The levels of TNF-α and IL-6 in the body after drinking alcohol can be used to determine whether liver injury has occurred.

In vitro cell experiments have found that the expression of TNF-α and IL-6 was elevated in alcohol-injured HepG2 cells, indicating that alcohol inducted severe inflammatory response. HFOPs + PL(1:1) pretreatment can significantly inhibit the expression of TNF-α and IL-6, indicating that HFOPs + PL(1:1) regulate inflammation by modulating the secretion of inflammatory factors. Its effectiveness is comparable to that of HFOPs.

Several peptides have also been affirmed to alleviate inflammation by regulating inflammatory factors. Through in vivo experiments, for example, Ganoderma lucidum peptides have been found to regulate inflammation by modulating the secretion of IL-6 and TNF-α. Black bean peptides have been found to prevent alcohol-induced liver injury by inhibiting IL-6 and IL-1 β [41].

In this study, high Fischer ratio oligopeptides (HFOPs) derived from *Chlamys farreri* and their preparations significantly lowered serum ALT and AST activities in mice. Furthermore, they markedly enhanced the activities of SOD and GSH-Px while reducing MDA levels compared to the alcohol model group. This alignment with the results of Zhao et al. supports the validity of our findings [36].

The mechanism by which HFOPs prevent alcohol-induced liver injury differs from that of phytochemicals, such as *Kaempferol*. Whereas kaempferol exerts its hepatoprotective effects primarily by directly inhibiting the expression and activity of CYP2E1 to reduce oxidative stress at its source [42], HFOPs functions through a more comprehensive modulation of the entire alcohol metabolism network.

Overall, HFOPs + PL(1:1) with half substitution of HFOPs by PL had the same anti-alcoholic and hepatoprotective effect with HFOPs, which has a certain protective effect on liver enlargement in alcoholic mice. However, the effect is less inferior to HFOPs and not significantly different from PL. Therefore, HFOPs + PL(1:1) as raw materials for the production of daily used antioxidant and hepatoprotective foods can effectively alleviate liver injury caused by long-term alcohol consumption, and the effect was better than that of glutathione, a commonly used drug in the market. The development of such alcohol-detoxified and hepatoprotective foods has a very broad market prospect.

## 5. Conclusions

In this paper, HFOPs with hepatoprotective against alcohol-induced injury were compounded with PL, a medicinal food component, and the anti-alcoholic and antioxidant capacities of HFOPs, PL, and HFOPs + PL(1:1) were evaluated by in vitro chemical methods and compared. The protective effects against alcoholic liver injury were further systematically investigated through in vitro cellular and in vivo animal experiments. Both HFOPs and PL have a promoting effect on ADH and SOD activities, with a dose-effect relationship within the concentration range of 1–5 mg/mL. HFOPs + PL(1:1) with half substitution of HFOPs by PL maintained the same ADH activity as HFOPs, had good antioxidant activity, and could reduce the damage caused by radicals. Then, an alcohol-injured HepG2 cell model was successfully constructed with 0.6 mol/L ethanol. HFOPs and PL had no toxic effects on HepG2 cells within the concentration range of 1–70 μ g/mL. On the one hand, it increased the content of antioxidant enzymes SOD and GSH in alcohol-injured HepG2 cells, reduced the generation of lipid peroxidation product MDA, inhibited alcohol-induced oxidative stress, and thus alleviated cell injury; on the other hand, it reduced the release of inflammatory factors TNF-α and IL-6 from the injured cells by inhibiting the cellular inflammatory response, playing an anti-inflammatory protective role. The effects of HFOPs, PL, and HFOPs + PL(1:1) on acute alcoholic liver injury in mice were further investigated and compared. It was found that the intake of HFOPs and HFOPs + PL(1:1) had the same hepatoprotective against alcohol-induced injury, both of which could effectively shorten the sobriety time with good anti-drunkenness effects, significantly enhance the activities of hepatic ADH and ALDH and reduce the levels of serum AST and ALT, which could accelerate the decomposition and metabolism of alcohol, and significantly reduce the degree of alcohol damage to the liver. It could also significantly enhance the activities of GSH-Px, SOD, and CAT in the antioxidant protection system and reduce MDA levels, demonstrating good antioxidant capacity, lowering lipid peroxidation product levels, and thus alleviating alcohol damage to the liver. Therefore, the HFOPs + PL(1:1) with half substitution of HFOPs by PL can not only maintain good hepatoprotective effect against alcohol-induced injury but also reduce the cost of production, which is of great significance in the development of anti-alcoholic and hepatoprotective products.

## Figures and Tables

**Table 1:** The drunkenness time, sobriety time, and liver index of drunken mice (X ± SD, *n* = 8).

Groups	Sample Concentration/(mg·mL^−1)^	Dose Administered/(mg·kg^−1^ bw)	Alcohol Dose/(mL·10g^−1^ bw)	Drunkenness Time/min	Sobriety Time/min	Liver Index/%
Normal	-	-	-	-	-	4.05 ± 0.09 ^e^
Model	-	-	0.09	10.13 ± 3.48 ^d^	65.38 ± 6.16 ^a^	5.23 ± 0.16 ^a^
GSH	5	50	0.09	14.13 ± 4.05 ^bcd^	58.13 ± 4.97 ^ab^	4.47 ± 0.12 ^bcd^
HFOPs	40	400	0.09	21.75 ± 4.33 ^ab^	48.88 ± 5.67 ^bcd^	4.49 ± 0.13 ^bcd^
PL	40	400	0.09	12.25 ± 4.68 ^cd^	52.25 ± 7.59 ^bc^	4.72 ± 0.21 ^bc^
HFOPs + PL(1:1)-L	20 + 20	200 + 200	0.09	19.63 ± 3.50 ^abc^	49.88 ± 7.68 ^bcd^	4.76 ± 0.14 ^b^
HFOPs + PL(1:1)-M	40 + 40	400 + 400	0.09	22.25 ± 6.54 ^ab^	44.75 ± 5.29 ^cd^	4.39 ± 0.28 ^cd^
HFOPs + PL(1:1)-H	60 + 60	600 + 600	0.09	25.13 ± 5.38 ^a^	39.63 ± 4.81 ^d^	4.34 ± 0.23 ^de^

Different letters in the same column indicate significant differences (*p* < 0.05).

## Data Availability

The original contributions presented in the study are included in the article, further inquiries can be directed to the corresponding author.
